# Direct Visualization of Cervical Interlaminar Epidural Injections Using Sonography

**DOI:** 10.3390/tomography8040157

**Published:** 2022-07-22

**Authors:** Nana Maeda, Manabu Maeda, Yasuhito Tanaka

**Affiliations:** 1 Maeda Orthopaedic Clinic, 864-1 Kidera-cho, Nara 630-8306, Japan; nana-k@mvd.biglobe.ne.jp; 2 Department of Orthopedics, Nara Medical University, 840 Shijo-cho Kashihara, Nara 634-8521, Japan; yatanaka@naramed-u.ac.jp

**Keywords:** ultrasound, epidural injection, cervical, steroid, superb microvascular imaging

## Abstract

In this case series, we describe a novel ultrasound (US)-guided cervical interlaminar epidural steroid injections (CILESIs) procedure that does not depend on the loss-of-resistance method for epidural space identification. A needle is introduced into three US-identified structures (triple bar sign), the interspinal ligament, ligamentum flavum, and dura mater. The injectants are monitored using superb microvascular imaging during injection. Here, we demonstrate the use of US-guided CILESIs in nine cases and propose the use of sonography, rather than conventional methods, for easier and safer cervical epidural injections. Sonography for direct visualization of cervical epidural injection may allow for outpatient injections.

## 1. Introduction

A major concern for cervical epidural injection (CEDI) in patients with neck pain and cervical radiculopathy is the safety and effectiveness of the procedure. Reports of serious complications such as spinal cord injury have led to concerns regarding this procedure’s safety [1,2,3]. Fluoroscopy or computed tomography (CT) guidance aims to reduce the risks of epidural injection, such as dural puncture or spinal cord injury. However, although both guidance approaches improve safety, they cannot completely aid in advancing the tip of the needle into the epidural space, as these approaches cannot visualize the entire intrusion route [2,3,4,5].

It is also important to examine the placement of the needle tip and the spread of the injectant to ensure its effectiveness [6]. The loss-of-resistance (LOR) method is performed for blinded or fluoroscopic epidural injections. However, it is limited by false-positive LOR before the needle enters the epidural space [7,8]. CT guidance uses high radiation exposure to carefully check the needle tip position, which also poses a problem [9]. However, CT cannot image the epidural space without a contrast medium, which can cause adverse effects [10]. Furthermore, CT emits ionizing radiation, which can cause cancer [11].

Sonography offers an alternative method for performing cervical interlaminar epidural steroid injections (CILESIs). It could help reduce the risk of injury, avoid the pitfall of false-positive LOR, and avoid radiation exposure or the use of contrast media by allowing direct visualization of the location of the needle tip during the procedure with cross-sectional imaging [6].

However, the technique for ultrasound (US)-guided CILESIs has not been reported, and no case series has described the safety of this technique in cervical lesions. Herein, we report cases of US-guided CILESIs without fluoroscopy.

## 2. Materials and Methods

### 2.1. Patients

This study was performed at the Maeda Orthopedic Clinic and was approved by its institutional review board (Commission of Ethics approval number 00000003).

Patients provided written informed consent for the cervical epidural block and the publication of this report. Overall, nine patients presented to the Maeda Orthopedic Clinic between August 2021 and January 2022. All patients underwent US-guided CEDI for the treatment of neck pain or interscapular radicular pain due to cervical disc herniation, cervical discopathy, cervical canal stenosis, or thoracic disc herniation.

### 2.2. US-Guided CEDI

**Figure 1:**
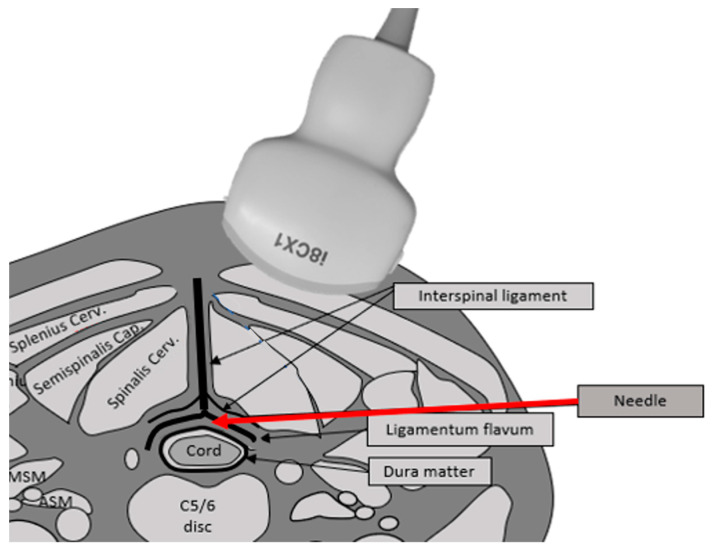
The illustration shows the needle insertion route on the cross-sectional image of the neck. The main structures are the cord, dura mater, interspinous ligament, and ligamentum flavum. The epidural space exists between the dura mater and the ligamentum flavum. The needle (red arrow) is introduced tangentially in between the ligamentum flavum and dura matter.

**Figure 2:**
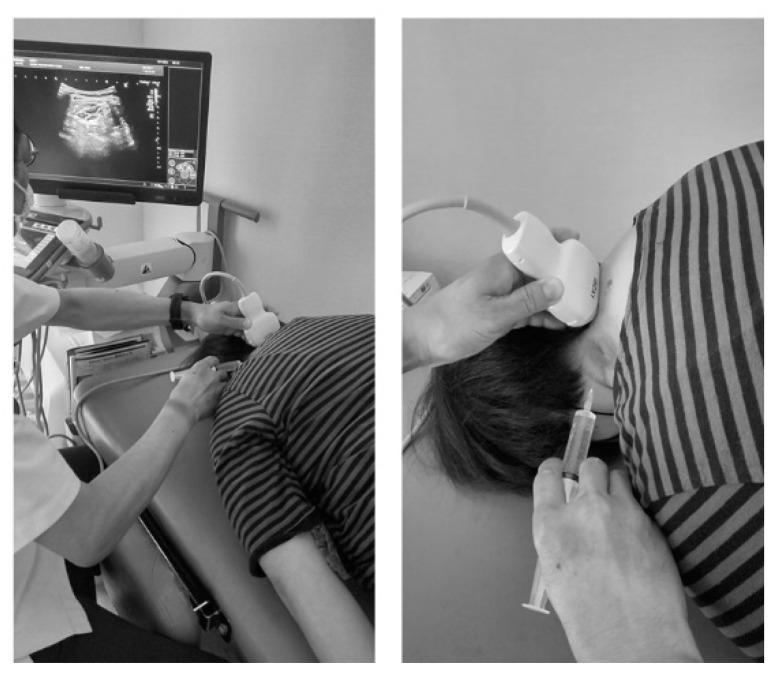
The patient is placed in the prone position, and the pillow is placed under the patient’s chest. Then, the patient is asked to place their forehead on the bed and flex their neck to the maximum extent possible.

**Figure 3:**
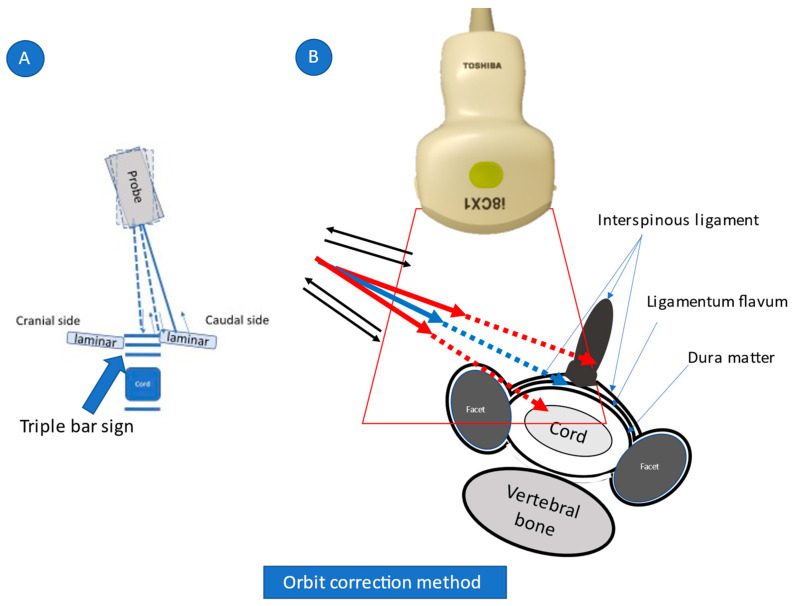
Orbit correction method.

This novel US-guided CILESIs was performed as follows. First, the axial image of the interspinal ligament, ligamentum flavum, and dura mater as a triple bar sign was identified using a posterolateral with transverse scan approach by a PVI-475BX convex probe (Aplio i800 systems; Canon Medical System, Tochigi, Japan) (Figure 1). To optimize this image, the patient was placed in the prone position, and a pillow was placed under their chest. The patient was then asked to place their forehead on the bed and flex their neck to the maximum extent possible (Figure 2). For needle placement, the tip of the needle was advanced into the ligamentum flavum, tangential to the dura mater, under US guidance using an in-plane technique (Figure 1, Video S1). At first, while inserting and removing the needle, the needle was translocated from the caudal (or cranial) side to the correct plane where the triple bar sign is seen clearly (Figure 3A; view from the side of the body). The needle direction should be corrected if needed. Red arrows indicate an inappropriate needle direction, with an extension line (indicated by dashed arrows) facing the spinal cord or the interspinal ligament. The needle trajectory should be corrected from those indicated by the red arrows to that indicated by the blue arrow (Figure 3B; view from the caudal side of the body). As no critical arteries or nerves are passed using this approach, the needle orbit correction can be repeated just before the needle tip reaches the ligamentum flavum. As a result, accurate needle placement is possible.

Video S1. The needle tip is advanced tangentially between the ligamentum flavum and dura matter under ultrasonography (US) guidance. This can be confirmed by the resistance of the needle tip when reaching the complex of the ligamentum flavum felt by the practitioner’s hand when checking the US monitor.

While being inserted and removed, the needle is translocated from the caudal (or cranial) side to the correct plane where a triple bar sign is seen clearly (Figure 3A). Next, the needle direction is corrected. Red arrows indicate inappropriate needle directions, with extension lines (indicated by dashed arrows) facing the spinal cord or interspinal ligament. The needle orbit should be corrected from the red to the blue trajectory (Figure 3B, view from the caudal side of the body). 

Using a posterolateral approach, the needle is visible from the insertion point to the epidural space between the ligamentum flavum and dura matter. At first, while being inserted and removed (indicated by blue arrows in the figure), the needle is translocated from the caudal (or cranial) side to the correct plane where a triple bar sign (which is composed of the interspinal ligament, ligamentum flavum, and dura matter) is seen clearly (Figure 3A; view from the side of the body). Next, the needle direction is corrected. Red arrows indicate inappropriate needle directions, with extension lines (indicated by dashed arrows) facing the spinal cord or interspinal ligament. The needle orbit should be corrected from the red to the blue trajectory (Figure 3B; view from the caudal side of the body). Because there are no critical arteries and nerves through this approach, the needle orbit correction can be repeated just before the needle tip reaches the ligamentum flavum. As a result, accurate injection is possible.

**Figure 4:**
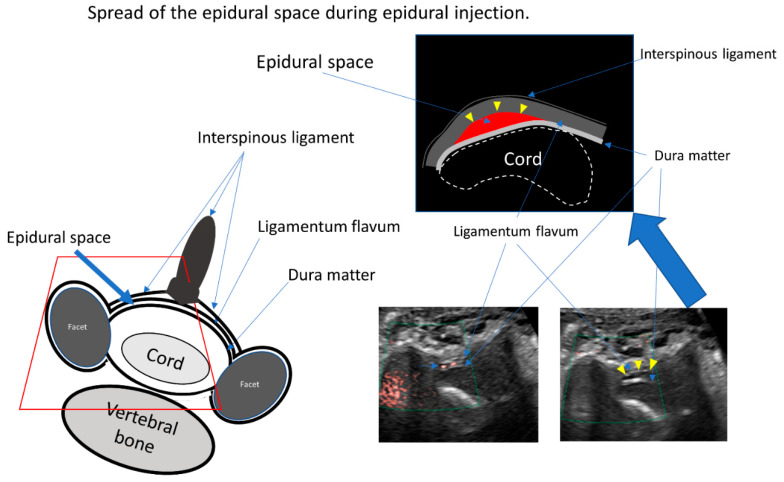
The illustration shows the cross section of the spinal canal. The main structures are the cord, the dura mater, the interspinous ligament, and the ligamentum flavum. The epidural space exists between the dura mater and the ligamentum flavum. The space of Okada exists between the interspinous ligament and the ligamentum flavum. Ultrasound data show the superb microvascular imaging signal during injection (left picture) and the enlargement of the epidural space after injection (upper illustration and right picture).

**Figure 5:**
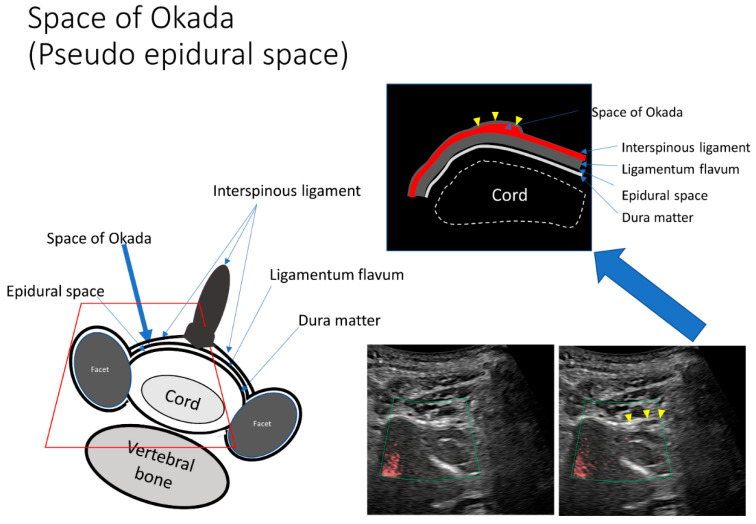
The pseudo-epidural space is enlarged by a small dose of injection. The space between the interspinous ligament and ligamentum flavum, such as the space of Okada, is observed sonographically. If the needle tip is located more superficially than the ligamentum flavum, the injectant will appear in the space of Okada or in the spinalis cervicalis muscle once administered.

Confirmation that the needle tip had reached the ligamentum flavum was achieved based on resistance according to the pressure felt by the operator whilst observing the US monitor. We then applied gentle pressure to the syringe plunger as the needle was slowly advanced into the space between the ligamentum flavum and dura mater reaching approximately two-thirds of the triple bar sign, where easy flow of the injectant was facilitated (Figure 4, Video S2). If the needle tip was located more superficially than the ligamentum flavum, any attempt to inject the injectant would be evident in the surrounding muscle tissue or the pseudo-epidural space, such as the space of Okada (Figure 5, Video S3).

Video S2. The needle is gradually advanced while applying pressure to the plunger. If the needle tip is located more superficially than the epidural space, microvascular imaging (SMI) shows that there is no flow of injectant into it. However, once the dense fibrous ligamentum flavum is pierced, the injectant flows. This is immediately confirmed using SMI. Subsequently, the epidural space is gradually enlarged by epidural injection.

Once the dense fibrous ligamentum flavum was pierced, the injectant was able to flow into the epidural space; this was immediately confirmed using superb microvascular imaging (SMI) (Figure 4, Video S2). Lastly, the practitioner verified the epidural space enlargement using the epidural injection. We used 4 mg of dexamethasone in 6 mL of 0.25% lidocaine for CEDI.

### 2.3. CT Epidurography

To confirm the accuracy of US-guided CEDI, we chose one patient (case 4) and two other patients (a 78-year-old male patient with cervical canal stenosis (Supplementary Materials S1) and a 58-year-old female patient with cervical disc herniation (Supplementary Materials S2) who were not included in this case series because they could not be followed up for >2 months). We performed US-guided CEDI at the C4/5 level. We used a contrast medium instead of dexamethasone (3.5 mL iohexol-240 [Omnipaque-240; GE Healthcare Pharma, Tokyo, Japan]) in 3.5 mL of 0.25% lidocaine. Immediately after injection of the contrast medium, anterior–posterior and lateral epidurograms of the cervical spine were captured. Subsequently, the patients were placed in the prone position within the gantry of the CT scanner (Aquilion Start Canon medical system, Tochigi, Japan) and CT was performed from C1 to L1 5 min after injection. The two orthopedic surgeons and the author have 32, 38, and 24 years of experience, respectively. Technical success was determined by reviewing CT epidurograms for the presence of epidural contrast. The spread of contrast within the epidural space was also assessed on CT epidurograms.

### 2.4. Pain Intensity

Pain intensity was evaluated using the visual analog scale (VAS). All patients were instructed on how to use the VAS prior to the cervical epidural block (0, no pain; 10, worst pain conceivable). Before the procedure, an interventional orthopedic doctor questioned the patients regarding their baseline VAS scores for pain. The patients were discharged on the day of the procedure and asked to revisit 1 week and 2 months later for evaluation of side effects and pain scores, respectively. If the pain persisted, we treated patients with an additional block and rechecked their pain scores.

### 2.5. Functional Ability

Functional ability was evaluated using the neck disability index (NDI). The NDI is a 10-item self-administered disease-specific questionnaire that evaluates the impact of neck pain on a patient’s daily life and the corresponding disability. The questionnaire scores range from 0 to 50; the higher the score, the greater the disability. The validity and reliability of this scale have been previously established [12].

Symptom improvement was assessed before and 2 months after the injection using the VAS and the Japanese version of the NDI. Any potential complications, including headache, increased neck pain, or stiffness, were monitored. Moreover, functional ability was rechecked in patients treated with an additional block.

Patients were asked about their opioid consumption, any additional cervical spine injections, and progression to surgery during the follow up period, and the answers were documented in their profile.

## 3. Results

During the study period, nine participants with radicular pain underwent US-guided CEDI. The radicular pain in one, two, three, and three patients was caused by thoracic disc herniation, cervical disc herniation, cervical discopathy, and cervical canal stenosis, respectively. These patients were followed up from 60 to 348 days after the procedure. Their baseline data are presented in Table 1. The baseline and demographic characteristics of the patients were recorded, and the mean age of patients was 51.2 years (range, 23–74 years). Six participants were men and three were women. The mean body mass index was 24.2 km/m^2^ (range, 19.3–31.2 km/m^2^). At the 2-month follow-up, there were significant reductions in NDI from 44.4 to 15.6 points in Table 2. The mean VAS score of neck pain was 8.3 at before the procedure and 1.8 at the 2-month follow-up in Table 2. Among those who reported having recurrent pain (four patients), no patient used opioids for analgesia, four patients reported receiving additional injections, and no patient underwent surgery. During the follow-up period, one patient was able to discontinue opioids.

Vascular or subdural injections were not confirmed in any of the patients during the procedure. Furthermore, we did not encounter any significant complications, including stroke or persistent neurological deficits, during the procedure or 2-month follow-up. Nausea and general fatigue were observed after epidurography using a contrast media (case 4); however, the symptoms resolved within a day.

CT epidurograms revealed that the procedure was technically successful in all three patients from whom they were obtained, including case 4. The spread of contrast was confirmed from the C2 vertebra level to the Th5 vertebra level according to the axial CT epidurogram.

SMI was performed with a small injection. An appropriate epidural space was detected using SMI after the needle was advanced. The epidural space was then enlarged by means of an additional injection directed to the area where the SMI signal was detected (Figure 4, Video S3).

Video S3. The retrodural space of Okada is gradually enlarged by epidural injection. This lesion is not a true space in the epidural space. The loss-of-resistance method cannot differentiate between the space of Okada and the epidural space.

### 3.1. Representative Cases

#### 3.1.1. Case 4

A 73-year-old woman presented with severe posterior cervical pain and right shoulder pain. The pain did not resolve with the use of medications (an opioid, an anti-inflammatory analgesic, and a muscle relaxant). Magnetic resonance imaging revealed cervical canal stenosis. Her pain persisted for 3 weeks; therefore, US-guided C3/4 interlaminar CEDI of 1 mL dexamethasone (4 mg) and 6 mL 0.25% lidocaine was administered. In the examination room, SMI was used to confirm that the parenteral solution had spread throughout the epidural space. Subsequently, the VAS score decreased from 10 to 5. An additional four blocks were performed for 2 months, and the VAS decreased from 5 to 2. 

**Figure 6:**
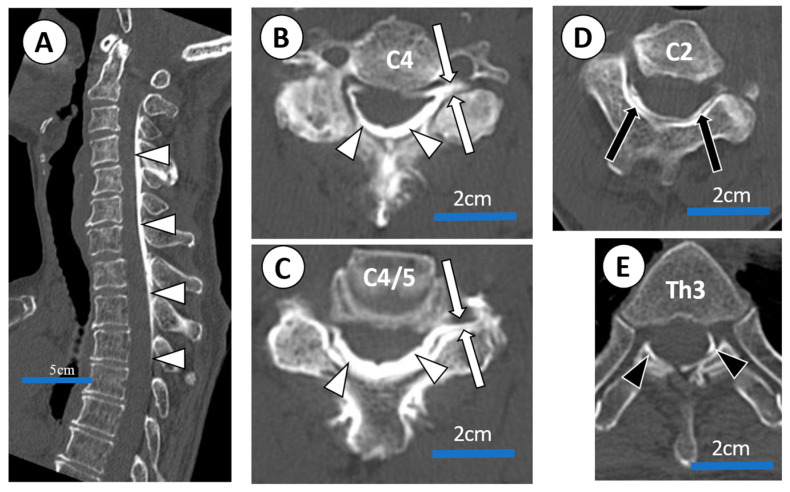
CT epidurogram after C4/5 US-guided epidural injection administered to a 73-year-old female with severe posterior cervical pain and right shoulder pain (case 4) associated with cervical canal stenosis. (**A**) Sagittal view of CT epidurogram. Dorsal spread (white arrow heads) between C2 and Th3 are confirmed. (**B**,**C**) Axial view of CT epidurogram (a slice of C4 vertebra and C4/5 disc; injection site). Dorsal spread (white arrow heads) and C5 root filling (white arrows) were confirmed. (**D**,**E**) Axial view of CT epidurogram at C2 and Th3 vertebra ). Cephalad spread (black arrows) was confirmed up to C2 vertebra level and caudal spread (black arrow heads) was confirmed down to Th3 vertebra level.

After 2 months, she experienced exacerbations and remission of symptoms. She received US-guided epidural blocks 20 times in total. We confirmed the 20th US-guided injection using CT epidurography (Figure 6).

#### 3.1.2. Case 5

A 73-year-old man presented with severe pain in his right shoulder and arm. Radiographs revealed that the patient had severe cervical spondylosis. Considering that severe night pain and insomnia had persisted for 2 weeks, US-guided C5/6 interlaminar CEDI of 1 mL dexamethasone (4 mg) and 6 mL 0.25% lidocaine was administered. Subsequently, the VAS score decreased from 10 to 1. No additional blocks were administered. No recurrence of pain was observed at the 8-month follow-up.

#### 3.1.3. Case 9

A 23-year-old man presented with severe pain in his right neck and interscapular region. The pain persisted despite diagnostic facet joint blocks (right Th5/6, right C3/4, and right C4/5 facet blocks). Magnetic resonance imaging revealed no vertebral bone injury; however, damage to the C3/4 and C4/5 discs was observed. The pain persisted for 3 months; therefore, US-guided C5/6 interlaminar CEDI of 1 mL dexamethasone (4 mg) and 6 mL 0.25% lidocaine was administered. In the examination room, SMI was used to confirm that the parenteral solution had spread throughout the epidural space. Subsequently, the VAS score decreased from 10 to 1. Two additional blocks were administered over 7 weeks, leading to the resolution of his pain. No recurrence of pain was observed at the 3-month follow-up.

## 4. Discussion

Reports of serious complications such as spinal cord injury have led to concerns regarding the safety of CEDI [1,2,3]. Epidural injections can be performed with or without a guide (blinded injection). Fluoroscopy or CT guidance aims to reduce the risks of epidural injection, such as dural puncture or spinal cord injury [13,14,15,16].

In the interlaminar approach, a guide is used to advance the needle tip just before the epidural space [13].

CT guidance in interlaminar ESI improves the evaluation of the proper positioning of the needle owing to its high resolution and cross-sectional imaging [13]. However, the introduction of the needle into the epidural space cannot be visualized according to the movement of the needle during the procedure. US, as an alternative method for performing CILESI, could help reduce the risk of injury by allowing direct visualization of the location of the needle tip during the procedure with cross-sectional imaging. However, a technique for US-guided CILESI has not been reported, and no case series have described its safety because US has some limitations in neuraxial (epidural or intrathecal) procedures as it has a limited resolution at deep levels and near bony surfaces that affect image quality, and it is not possible to visualize the real-time propagation of the injectable in the epidural or intrathecal space [17].

CEDI is based on the LOR method [2,7]. The epidural space is closed by negative pressure unless there is an abscess or bleeding. It is approximately 1–2 mm in diameter at levels above C6-C7 and is less dependent on individual variation, including the presence of spinal stenosis [14,15]. Thus, the safety zone for interlaminar CEDI is very narrow [16]. In reviews of malpractice claims between 2005 and 2008, 64 cases involved cervical interventions, 20 of which resulted in direct spinal cord injury associated with interlaminar CEDI [5,9].

The needle tip position must be visualized throughout the procedure to ensure accurate injection into the epidural space. Clinical practice guidelines recommend fluoroscopic guidance in all CEDIs [18]. However, fluoroscopic guidance methods are limited because they use bones as landmarks. Therefore, it is difficult to guide the needle tip into the gap of the ligamentum flavum and dura mater safely, as it is approximately 1 mm thick and permeable to X-rays, without direct visualization.

For the accurate insertion of the needle, the US monitor, the affected area, and the practitioner should be in a straight line. However, this is not always possible during outpatient examinations because the size of the examination room is limited. Furthermore, since the needle is inserted deeply, needle orbit correction is often needed. As there are no critical arteries or nerves beyond the ligamentum flavum through this approach, the needle orbit correction can be repeated just before the needle tip reaches the ligamentum flavum. As a result, accurate injection is possible with our US guided method.

The LOR technique relies on penetration of the ligamentum flavum. However, up to 74% of the ligamenta flava are discontinuous in the midline of the cervical region [19]. Dural puncture cannot be prevented based on bony landmarks or the LOR technique because the needle is inserted near the midline under conventional method. Furthermore, the safety of the CEDI procedure is highly dependent on the operator’s experience. In contrast, US can easily identify the ligamentum flavum, dura mater, and the needle tip independent of the operator’s experience.

If the needle is advanced deeply, dural puncture or spinal injury can be avoided because the needle is tangentially advanced with respect to the dura. Even in cases of inappropriate injection, the practitioner could use the US image to confirm and terminate the inappropriate injection from the beginning, if necessary. Therefore, serious complications, such as total spine anesthesia or spinal injury, could be avoided. The direction of the needle has two advantages. The first is improved needle visibility by bringing the needle closer to the probe in parallel. The second is the avoidance of dural puncture and spinal cord injury.

**Figure 7:**
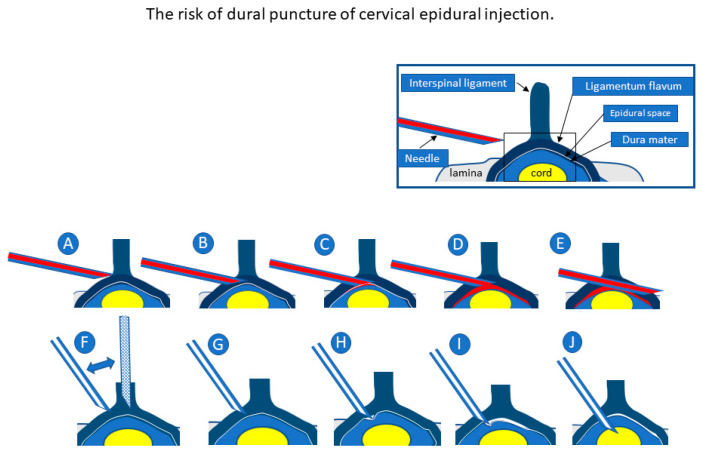
Upper figures show our cervical epidural injection (CEDI) method in which the needle is inserted tangentially into the dura (**A**–**E**). Lower figures show the conventional CEDI method wherein the needle is inserted nearly perpendicular to the dura (**F**–**J**). In both methods, loss of resistance may be confirmed before and after the insertion into the flavum (**A**,**C**,**F**,**H**). The closer the needle is inserted vertically, the higher the risk of dural puncture because the safety margin between the flavum and the dura becomes narrower (**G**,**H**). The risk of dural puncture can be avoided if the needle is inserted tangentially into the dura (**C**,**D**). In the conventional method (**J**), when the needle is advanced deeply, dural puncture cannot be avoided. Meanwhile, in our method, dural puncture can be avoided because the needle penetrates tangentially to the dura (**E**). The flow of the injectant into the epidural space is visualized in real-time under ultrasound-guided epidural injection (**C**).

Unlike US guidance, fluoroscopy and CT guidance (Figure 7) cannot accurately visualize the needle tip position. Fluoroscopy-guided CEDI can only confirm whether the LOR method is performed correctly based on contrast, identifying the epidural space after the trial of LOR. Additionally, CT-guided CEDI cannot show needle movement in real-time as the procedure progresses. The placement of the needle tip is only confirmed after needle insertion. SMI methods have been used to identify slow blood flow in the body. Unlike CT and fluoroscopy, SMI helps visualize not only microvessels with slow blood flow but also the movement of liquid (injectant) flow without contrast injection [20], and it can be a useful tool to confirm if the needle tip is in proper anatomical position e.g., the epidural space. Under SMI, the spread of the injectant can be visualized using US while simultaneously confirming the proper placement of the tip of the needle between the ligamentum flavum and dura matter (Figure 3) [6]. The major difference is that it offers real-time, radiation-free guidance for interventions.

The LOR method can also be used to precisely detect the epidural space using SMI signals. However, the LOR method is associated with false-positive LOR before the needle enters the epidural space. False-positive LOR occurs in 29.4% of patients who undergo CEDI under conventional fluoroscopic guidance [7]. The retrodural space of Okada is a potential space dorsal to the ligamentum flavum that allows communication between the bilateral facet joints and interspinous bursa [8,21,22]. In the current study, the space of Okada on the ligamentum flavum was visible using US (Figure 5, Video S3).

US guidance allows better positional relationships between the needle tip and anatomical structures (e.g., lamina, space of Okada, and epidural space) in cross-sectional imaging such as CT guidance. A previous investigation examining conventional fluoroscopy-guided lumbar interlaminar ESI demonstrated that many non-target injections in the retrodural space of Okada are likely to go unnoticed at the time of the procedure [21]. This may be because conventional fluoroscopy contrast material in the retrodural space of Okada can mimic true dorsal epidural spread as these two spaces run parallel to each other and may therefore overlap in standard lateral and anteroposterior projections [21,22]. Our US data also revealed that the space is too close to be identified by fluoroscopy. This suggested that inappropriate injection by LOR could be avoided using US.

Riveros-Perez et al. introduced color Doppler imaging to confirm the correct position of the epidural needle in the lumbar spine [23]. However, this method has not been applied to CEDI [17]. For cervical epidural injection, Zhang et al. reported transforaminal epidural steroid injections using sonography [24]. However, they did not employ color Doppler imaging to confirm the correct position of the needle and neither did they visualize the spinal cord, its nutrient vessels, or the radicular artery during the procedure. Serious complications such as spinal cord injury, either directly or indirectly via injury of its nutrient vessels, have been reported in transforaminal epidural steroid injections [1,2,3]. We visualized the spinal cord and surrounding vessels during the interlaminar epidural injection using the SMI method. To the best of our knowledge, our study is the first to achieve direct visualization of not only the entry route of the needle but also of the spinal cord and surrounding vessels during the CILESIs procedure.

## 5. Conclusions

In conclusion, we propose the possible use of US for easier and safer CILESIs compared with conventional methods. US for the direct visualization of CILESIs may allow for safer outpatient injections.

## Figures and Tables

**Table 1:** Clinical and demographic characteristics.

Case	Age (years)	Sex	Body Mass Index (kg/m^2^)	Diagnosis
1	24	M	24	Th1/2 Disc herniation
2	45	M	31.2	C2/3, 5/6, 6/7 Disc herniation
3	51	M	20.9	Cervical discopathy
4	73	F	19.3	Cervical canal stenosis
5	73	M	24.7	Cervical canal stenosis
6	63	F	28.8	C5/6 Disc herniation
7	74	F	21.4	Cervical canal stenosis
8	35	M	26.2	Cervical discopathy
9	23	M	21	Cervical discopathy

**Table 2:** Pain intensity and functional ability before and after ultrasound-guided epidural injection.

Case	Injection Site	Pre Visual Analog Scale Scores	Post Visual Analog Scale Scores	Neck Disability Index	Additional Injection (Times)	Additional Opioid Consumption (Times)
1	C6/7	10	2.5	40%→4%	0	0
2	C5/6	10	5	70%→26%	0	0
3	C5/6	4	2	16%→20%	0	0
4	C4/5	10	2	94%→34%	20	0
5	C5/6	5.9	0	8%→2%	0	0
6	C4/5	9	0	48%→10%	0	0
7	C3/4	10	5	54%→28%	4	0
8	C6/7	9	0	28%→4%	4	0
9	C5/6	6.5	0	42%→12%	2	0

## Data Availability

The data that supports the findings of this study are available in the supplementary material of this article.

## Supplementary Materials

The following are available online at https://www.mdpi.com/article/10.3390/tomography8040157/s1, Figure S1: a 78-year-old male patient with cervical canal stenosis, Figure S2: a 58-year-old female patient with cervical disc herniation, Video S1: Approach of US guided cervical epidural injection, Video S2: Enlargement of the space of OKADA, Video S3: Spread of the epidural space during epidural injection.
