# Supplementary figures and images for: Direct Visualization of Cervical Interlaminar Epidural Injections Using Sonography
Source: Tomography. 2022 Jul 22;8(4):1869–80. doi: 10.3390/tomography8040157 (PMC9341393; doi:10.3390/tomography8040157)

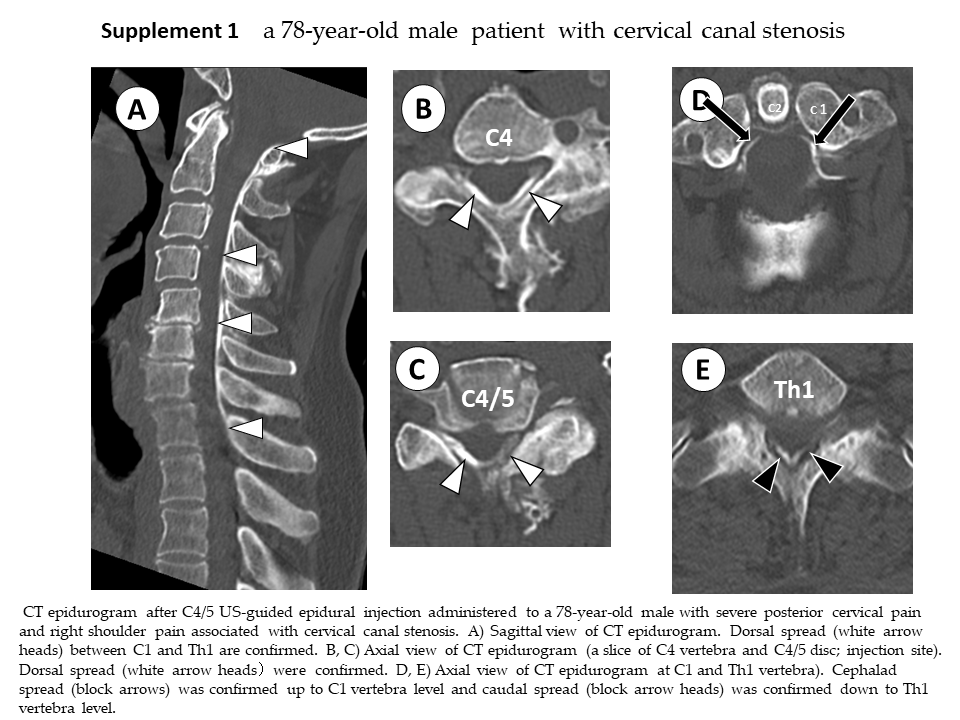

Supplement: Supplementary file 1 [file tomography-08-00157-s001.zip › Supplement Figure S1.tif]

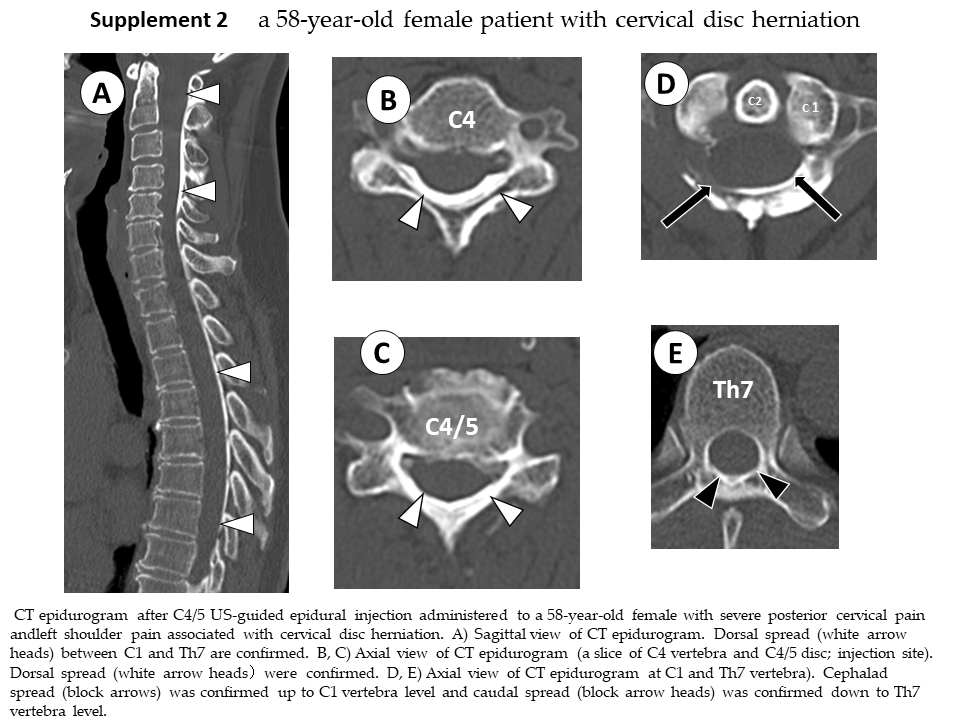

Supplement: Supplementary file 1 [file tomography-08-00157-s001.zip › Supplement Figure S2.tif]
